# Development of Primer Pairs from Molecular Typing of Rabies Virus Variants Present in Mexico

**DOI:** 10.1155/2016/4659470

**Published:** 2016-08-03

**Authors:** Fernando Bastida-González, Dolores G. Ramírez-Hernández, Erika Chavira-Suárez, Eleazar Lara-Padilla, Paola Zárate-Segura

**Affiliations:** ^1^Laboratorio de Medicina Traslacional, Escuela Superior de Medicina, Instituto Politécnico Nacional, Plan de San Luis y Díaz Mirón s/n, Santo Tomás, Miguel Hidalgo, 11340 Ciudad de México, CDMX, Mexico; ^2^Laboratorio de Biología Molecular, Laboratorio Estatal de Salud Pública del Estado de México, Paseo Tollocan s/n, La Moderna de la Cruz, 50180 Toluca, MEX, Mexico; ^3^Departamento de Bioquímica, Facultad de Medicina, Universidad Nacional Autónoma de México, Avenida Universidad 3000, Copilco Universidad, 04510 Coyoacán, CDMX, Mexico

## Abstract

Nucleoprotein (N) gene from rabies virus (RABV) is a useful sequence target for variant studies. Several specific RABV variants have been characterized in different mammalian hosts such as skunk, dog, and bats by using anti-nucleocapsid monoclonal antibodies (MAbs) via indirect fluorescent antibody (IFA) test, a technique not available in many laboratories in Mexico. In the present study, a total of 158 sequences of N gene from RABV were used to design eight pairs of primers (four external and four internal primers), for typing four different RABV variants (dog, skunk, vampire bat, and nonhematophagous bat) which are most common in Mexico. The results indicate that the primer and the typing variant from the brain samples, submitted to nested and/or real-time PCR, are in agreement in all four singleplex reactions, and the designed primer pairs are an alternative for use in specific variant RABV typing.

## 1. Introduction

Despite the significant progress for prevention of the rabies disease and its control in the developing countries, this disease still causes over 60 thousand human deaths every year. Rabies disease is caused by infection with viruses of the family Rhabdoviridae, genus* Lyssavirus* [1]. Until now, fourteen species of* Lyssavirus* have been described in the world. Actually, the* rabies virus* (RABV) is the only one present in the American continent [2–6].

Although all mammals are susceptible to lyssaviruses, bats and carnivores are the major* Lyssavirus* reservoirs. In the Americas, distinct RABV variants are associated with different animals, such as foxes, coyotes, raccoons, skunks, and multiple species of nonhematophagous (frugivorous, insectivorous) and hematophagous bats [7–12]. In Mexico, we have been faced with less than ideal surveillance in animal populations. The reduced resources available are prioritized for diseases with overwhelming human morbidity and mortality. Accurate diagnosis and determination of RABV variants are paramount components of surveillance system and frequently are important from the perspective of veterinary and public health, when the source of exposure needs to be determined and relevant control strategies need to be implemented [13].

The direct fluorescent antibody test (FAT) is the “gold standard” for rabies diagnosis [14]; the modern conjugates used in FAT are able to detect antigens of all lyssaviruses described to date [15, 16]. Virus variants associated with certain host species can be distinguished by application of anti-nucleocapsid monoclonal antibodies (MAbs) via indirect IFA. The MAbs are still commonly used in Latin American countries, particularly in the laboratories lacking established molecular techniques [17]; these have been applied to Mexican rabies virus samples and provide data regarding the most likely reservoir species involved in rabies transmission and dissemination. Even though there has been a decrease in dog rabies, as a result of massive dog vaccination in Mexico, there is a high risk of an increase of human rabies cases transmitted from wild reservoirs as well as the simultaneous presence of more than one reservoir and more than one virus variant [18–20].

The reactivity of certain viral isolates does not match the reactivity patterns in some cases [8, 17–19]. Other molecular assays like the restriction analysis of RT-PCR amplified fragments of RABV genes were suggested for the differentiation of two major RABV variants but suffered from low specificity. Amplification and sequencing of viral genes followed by their phylogenetic analysis have provided more robust characterization. However, this approach requires expensive equipment and experienced laboratory staff, and it takes a relatively long processing time (typically at least 10–12 hours) [20–23]. So, in this study, we designed eight pairs of primers of the RABV associated variant, which were used in a nested endpoint RT-PCR (four external and four internal primers) for the real-time RT-PCR assay, in order to detect and type the major RABV variants present in Mexico.

## 2. Materials and Methods

### 2.1. Primer Design

Primer design was based on the alignment constructed with ClustalW using complete RABV N gene sequences available in GeneBank associated variant; these were designed in consensus region. Two pairs of the primers (external and internal) were designed for each of the RABV variants associated, and the maximum average entropy (Hx) and the maximum entropy of each position were calculated using Bio Edit v7.2.5.

Two external primers and two internal primers were designed for dogs variant; 36 N gene sequences were obtained from different Mexican states; for the vampire bats variant, 18 N gene sequences were considered from Mexican states; for the nonhematophagous bat variant, the primer design comprised 50 N gene complete sequences from hosts* Eptesicus*,* Myotis*, and* Nycticeius* genera, distributed close to Mexico [21]; these genera are distributed from North America to Central America and have high diversity; in the case of skunks variant, 4 Mexican RABV sequences were considered; 34 RABV sequences were from USA and 13 CASK RABV sequences were from USA related to Mexican skunk rabies virus; previous studies consider two variants circulating in Mexico, MEXSK-2 and MEXSK-1 [22], located in South Baja California (SBC skunk) and Central Mexico; these are closely related and circulate predominantly in spotted skunks [23] (Table 1).

### 2.2. Samples

Twenty-three brain samples collected in Mexico were used as follows: nine brain samples tested negative by FAT and fourteen tested positive by FAT and typed by MAbs: these RABV isolated consisted of six samples of dog brain, one sample of skunk, two samples of cow, two samples of vampire bat, and three samples of nonhematophagous bat (Table 2).

### 2.3. Nucleic Acid Extraction

The brain tissues (approximately 3 mm^3^) were homogenized in 200 *μ*L of lysis/binding buffer using MagNA Lyser Green Beads (Roche, Germany) and MagNA Lyser (Roche Applied Science, Germany). Total RNA was extracted from the homogenates using MagNA Pure LC Total Nucleic Acid Isolation kit (Roche, Germany) and MagNA Pure LC 2.0 (Roche Applied Science, Germany) following the manufacturer's instructions. Total RNA was eluted in 200 *μ*L buffer elution and quantified into Nanodrop (Invitrogen); RNA concentration was calculated considering 1 UAb_*λ*260 nm_ = 50 ng/*μ*L, and all samples were adjusted at 20 ng/L of final concentration with elution buffer.

### 2.4. Nested RT-PCR Amplification and Sequence Determination

The reverse transcription reaction was performed using four singleplex reactions with external primers and SuperScript®III Platinum® One-Step qRT-PCR kit (Invitrogen) in 50 *μ*L of reaction mixture containing 25 *μ*L of 2x reaction mix, 1 *μ*L of forward sense primer (10 *μ*M), 1 *μ*L of reverse sense primer (10 *μ*M), 1 *μ*L SuperScript III RT/Platinum TaqMix, 17 *μ*L of DEPC-treated water, and 5 *μ*L RNA extracted (20 ng/*μ*L). Amplification was performed in C1000 Thermal Cycler (Bio-Rad, USA) using the following program: one cycle of RT at 50°C for 30 min, followed by denaturation at 92°C for 3 min, 35 cycles with denaturation at 92°C for 30 s, annealing at primer-specific temperature (Table 3) for 30 s, and elongation at 72°C for 1 min, with the final extension at 72°C for 4 min.

For nested PCR, 1 *μ*L of the primary amplification products was added to a new singleplex PCR reaction using internal primers and Taq DNA polymerase kit; in a 50 *μ*L total volume, add 5 *μ*L 10x PCR buffer, 1 *μ*L of 1x dNTP mix (200 *μ*M of each dNTP), 1 *μ*L internal forward primer, 1 *μ*L internal reverse primer, 0.25 *μ*L* Taq* DNA polymerase (1.25 units/reaction), 1 *μ*L of primary amplification, and 40.75 *μ*L RNase-free water. The thermal program consisted of a first cycle of 2 min at 94°C, followed by 35 repetitive cycles of denaturation of 1 min at 93°C, 1 min of annealing at the primer-specific temperature (Table 3), 1 min of elongation at 72°C, and the final elongation at 72°C for 4 min. The four singleplex RT-PCR and four nested PCR products were analyzed in 1-2% agarose gel. Bands of the expected size were excised, purified, and cloned in TOPO-TA vector (Invitrogen, Carlsbad, USA). The resulting plasmids were purified from* E. coli* colonies using Pure Link*™* Quick Plasmid Miniprep kit (Invitrogen), sequenced with the universal M13 primers (Macrogen, Korea), and analyzed with MEGA 6.06 [24].

### 2.5. Real-Time RT-PCR with SYBR Green

The one-step real-time PCR was performed using internal primers and LCFastStart RNA Master SYBR Green I kit (Roche, Germany) in 20 *μ*L of total volume, four singleplex reactions including 100 ng total of total RNA and 0.01 *μ*M of each internal pair of primers for RABV associated variant (Table 3). Amplification was performed in LightCycler 2.0 (Roche, Germany) using the following program: one cycle of RT at 55°C for 30 min, followed by denaturation at 95°C for 30 s, 40 cycles with denaturation at 95°C for 10 s, annealing at 60°C for 15 s, and elongation at 72°C for 25 s. The measurement of the fluorescent signal was carried out during the extension phase at 530 nm. By the end of the amplification test, an analysis of the dissociation curves from the product was made to ensure the absence of hairpin and dimer formation. Hybridization temperatures and primer concentrations were optimized for each reaction based on the preliminary standardization experiments.

## 3. Results

The set of primers for specific RABV variants was designed aligning the sequence of N gene region. Four regions highly conserved were selected for two external primers and two internal primers designed, with more than 90% of conservation, for each variant and high variability between variants. Two mismatches were permitted for the primers design, and less was possible for the internal primers located at the primers beginning or end.

**Figure 1:**
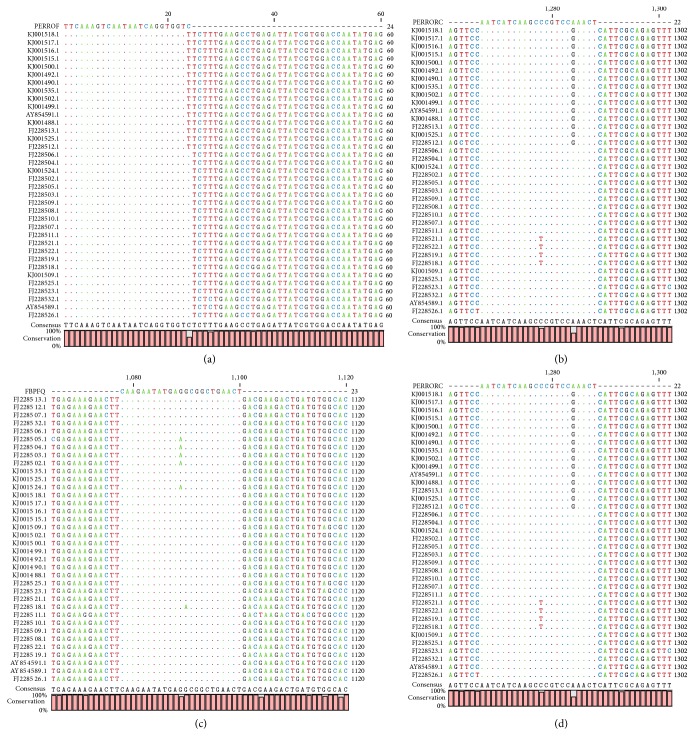
External and internal primer alignments for dog specific RABV variant detection. (a) Forward sequence of the external primer named PERROF. (b) Reverse sequence of the external primer named reverse PERRORC. (c) Forward sequence of the internal primer named FBPFQ. (d) Reverse sequence of the internal primer named PERRORC.

**Figure 2:**
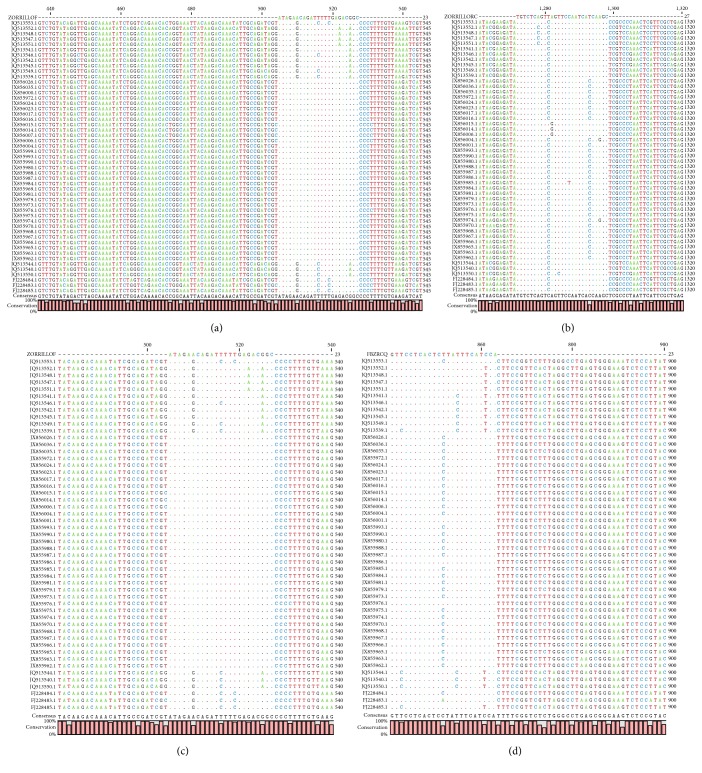
The external and the internal primer alignments for skunk specific RABV variant detection. (a) Forward sequence of the external primer named ZORRILLOF. (b) Reverse sequence of the external primer named ZORRILLORC. (c) Internal primer named ZORRILLOF. (d) Reverse sequence of the internal primer named FBZRCQ.

**Figure 3:**
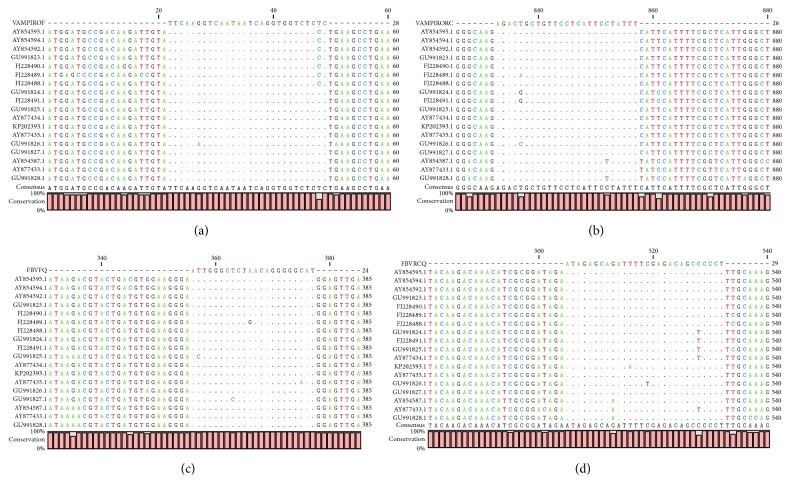
The external and the internal primer alignments for vampire bat specific RABV variant detection. (a) Forward sequence of the external primer named VAMPIRORC. (b) Reverse sequence of the external primer named VAMPIROF. (c) Forward sequence of the internal primer named FBVFQ. (d) Reverse sequence of the internal primer named FBVRCQ.

**Figure 4:**
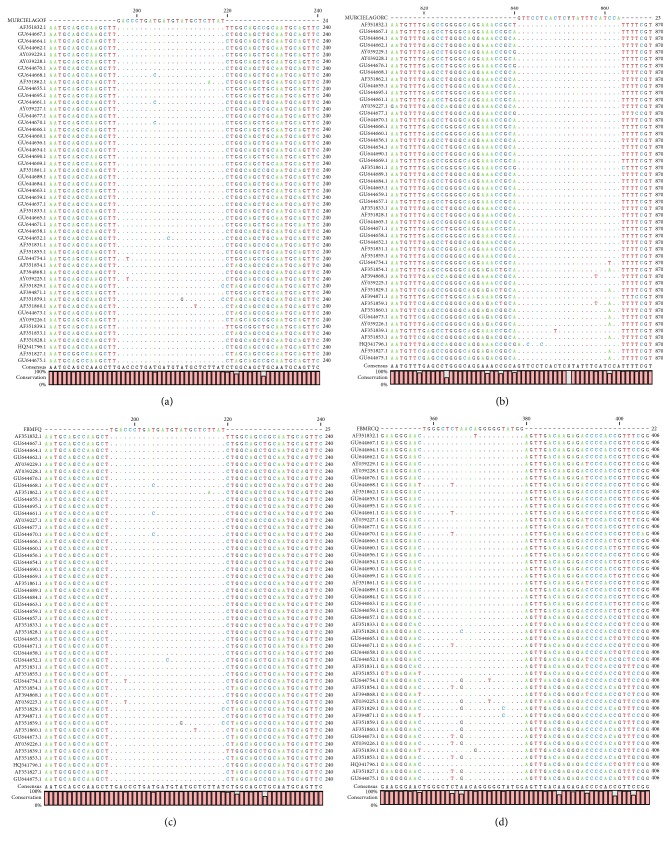
The external and the internal primer alignments for nonhematophagous bat specific RABV variant detection. (a) Forward sequence of the external primer named MURCIELAGOF. (b) Reverse sequence of the external primer named MURCIELAGORC. (c) Forward sequence of the internal primer named FBMQ. (d) Reverse sequence of the internal primer named FBMRCQ.

As the maximum entropy values increased, the number of identified conserved regions, their length, the coverage of conserved regions, and the average length of single conserved regions also increased. Two external primers and two internal primers were designed for dog-associated variant; the alignment presented a high conservation level (Figure 1). The maximum average entropy (Hx) was 0.04 and the maximum entropy of each position was 0.97. In the case of the set of primers for skunk-associated variant, the alignment presented high conservation level (Figure 2). The maximum average entropy (Hx) was 0.19 and the maximum entropy of each position was 0.99. The alignment of the set of primers for vampire bat-associated variant showed a high conservation level (Figure 3). The maximum average entropy (Hx) was 0.04 and the maximum entropy of each position was 0.98. Finally, the alignment of the set of primers for nonhematophagous bat-associated variant presented high conservation level (Figure 4). The maximum average entropy (Hx) was 0.07 and the maximum entropy of each position was 0.97 on average; this means that, at the same position of every base, a few sequences of alignment of the associated variant differed from the others and thus were considered conservative; with respect to the maximum average entropy, the variant associated with more differences was the nonhematophagous bat-associated variant (Figure 4).

The sequences and locations of the two pairs of variant-specific primers are listed in Table 3. Even when the melting temperature is similar between them, the sequence is dependent on the specific host variant.

**Figure 5:**
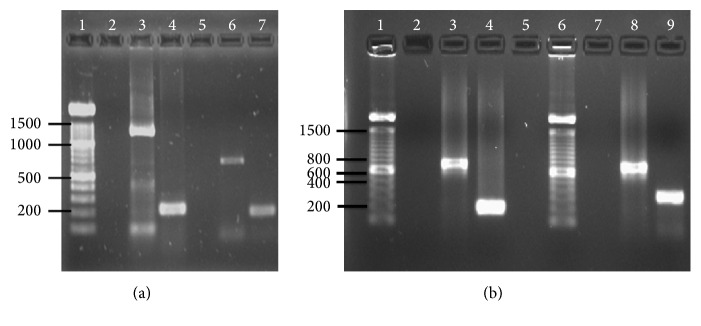
Mammals specific rabies virus variants detected by nested endpoint PCR assays. (a) Detection of dog specific variant (lines 2–4) and bat specific variant (lines 5–7). Lines 2 and 5: brain negative sample; lines 3 and 6: external amplification by RT-PCR (1187 and 668 pb, resp.); lines 4 and 7: internal amplification by nested PCR. (b) Detection of vampire bat specific variant (lines 2–4) and skunk specific variant (lines 7–9). Lines 2 and 7: brain negative sample; lines 3 and 8: external amplification by RT-PCR (835 and 795 pb, resp.); lines 4 and 9: internal amplification by nested PCR; line 5: empty. Lines 1 in (a) and 1 and 6 in (b) correspond to 100 pb DNA-ladder.

All brain samples from Mexican host mammals were diagnosed as negative or positive in FAT, with a corresponding signal in the nested RT-PCR assay. A positive control for each variant was performed using a positive example previously MAbs tested. In the first step, the external amplification produced a single band of 608–1187 bp, while the second amplification of the primary PCR products with the internal primer showed products of 200–400 bp. To complement the nested information, one-step RT-PCR as well as the second nested RT-PCR was performed with external primers and the same samples (Figure 5).

The optimal annealing temperature for external RT-PCR was in the range 48–55°C and was 56°C in the case of nested RT-PCR. Optimal concentration of Mg2+ was in the order of 2.5–3 mM for both RT-PCR and nested RT-PCR reaction mixtures.

### 3.1. RT-PCR SYBR Green

**Figure 6:**
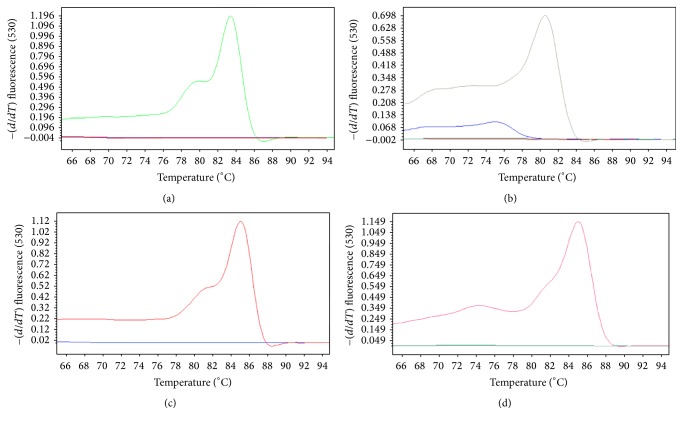
Specific rabies virus variants detected by real-time RT-PCR assays. (a) Bat specific variant. (b) Dog specific variant. (c) Vampire bat specific variant. (d) Skunk specific variant.

For real-time RT-PCR assay, we used the internal primers (Table 3). This technique had an optimal annealing temperature of 60°C from four pairs of primers, and the dissociation temperature curves were as follows: 85.50°C for skunk specific variant, 80.19°C for dog-associated variant, 83.96°C for bat-associated variant, and 85.23°C for vampire bat specific variant (Figure 6).

### 3.2. Comparison of Diagnostic Methods

A total of twenty-three samples were assessed as follows: nine negative-control samples performed by nested or real-time RT-PCR assays showed no positive detection with the internal and external primers; previously, fourteen positive-RABV variants samples were tested by FAT; eleven of them were categorized with monoclonal antibodies resulting in six positive to variant 1 (V1), three to V8, one to V5, two atypical variants, and two undetected. The RABV specific variant characterizations to dog, vampire bat, nonhematophagous bat, and skunk were determined by real-time RT-PCR using external primers. However, to prevent cross-reaction and to increase sensitivity in the nested PCR, internal primers were used to confirm thus the abovementioned variants. In addition, real-time PCR detection using internal primers confirmed the reservoir variant with dissociation temperatures of 60°C. The amplified fragments were sequenced with a subsequent analysis by BLAST; this analysis confirmed the reservoir for nested PCR and real-time RT-PCR (Table 4).

### 3.3. Sensitivity of nRT-PCR and SYBR Green

Twenty-three brain samples were analyzed as follows: nine negative-control samples and fourteen positive samples were confirmed by nucleotide sequencing. Regarding the results of the nested PCR and real-time PCR assays of the brain samples, they showed 100% sensitivity (100% CI: 76.84% to 100.00%) and 100% specificity (100% CI: 66.37% to 100%).

## 4. Discussion

In some studies of antigenic characterization of rabies virus, a panel of eight anti-N protein monoclonal antibodies (MAbs) has been used, which can differentiate between eleven distinct variants harbored by a variety of terrestrial and chiropteran hosts [25, 26]. Application of this panel to rabies virus collections from many Latin American countries has identified two major variants, associated with dog and vampire bat* (Desmodus rotundus)*, as well as other variants associated with several insectivorous bats, including the free-tailed bat* (Tadarida brasiliensis)* and the hoary bat* (Lasiurus cinereus)* [27].

Real-time RT-PCR techniques have been used for diagnosis and genotyping of all the* Lyssavirus* genus including the RABV [28]. The nested RT-PCR assay, which requires both multiple transfers of material and substantial time, is sufficient to detect virus from each virus-positive brain sample [29] and therefore still offers a useful tool for variants rabies diagnosis where conventional PCR technology exists.

The real-time RT-PCR detection with SYBR Green, where the specificity is being given by the primers, is an easy-to-use assay to detect infected brain material in a single tube test and, consequently, is an attractive option for laboratory use as a screening surveillance tool. In the present study, these latest technologies for typing the RABV variants depending on the host (vampire bat, skunk, dog, and bat) were used.

The real-time RT-PCR detection with SYBR Green, whose specificity is given by the primers, is an easy-to-use assay to detect infected brain material in a single tube test and, consequently, is an attractive option for laboratory use as a screening surveillance tool. In the present study, these latest technologies for typing the RABV associated variants on the host (vampire bat, skunk, dog, and bat) were used.

In the design with highly specific primers from the conserved region from the nucleoprotein of RABV, the maximum average entropy (Hx) was in the order of 0.03–0.19 and the maximum entropy of each position was 0.97–0.99. In addition, the positions of different primers in N gene sequence are close but different for variant host, increasing the specificity.

The current gold standard test has been and is the fluorescent antibody test (FAT), which uses a conjugated monoclonal antibody against the RABV nucleoprotein. Although it is cheaper, some laboratories have no access to MAbs but have PCR and/or real-time technology.

In the characterization of the antigenic variants (AgV) with MAbs in the dog samples, the dog variant-specific primers identified the dog variant (V1). This result matched both the nRT-PCR and SYBR Green primers at 100%. Similarly, the skunk samples matched the same percentage with skunk variant-specific primers. Furthermore, in the bovine samples where the MAbs detection identified the skunk variant (V8), the determined host by nRT-PCR and SYBR Green was diagnosed as positive with the vampire primers, with this last result confirmed by sequences and AC Numbers JQ037818 to JQ037831 (Table 1). The real-time RT-PCR result coincides with some other studies where rabies transmission from vampire bats to bovines has been described.

The MAbs detection in nonhematophagous bat was V5 bat, a result which coincides with both nRT-PCR and SYBR Green with the bat primer. The vampire bat 110 sample was determined as atypical and the vampire bat 3919 was not determined with the MABs; however, both samples were diagnosed as positive with the bat primers for nRT-PCR and SYBR Green.

In some cases, the classification of certain rabies virus isolates by monoclonal panel can obtain nontypical reactivity patterns and is not assigned to any known variant, as found in certain Argentinian rabies viruses [12]. The application of molecular genetic techniques for characterization of viral collections can assist in resolving such typing difficulties.

In the hematophagous bat samples determined as atypical and the one not determined with the MAbs, it was concluded that the host was a vampire bat by nRT-PCR and SYBR Green detection. This may have occurred due to the high sensitivity of the RT-PCR molecular technique, as it has been shown in studies where positive results in brains analysis were demonstrated by nRT-PCR and negative results by FAT [30, 31]. In all results, the host was confirmed by the amplicon sequencing. The access numbers are shown in Table 4.

According to the RABV variant detection, the external primers and internal primers detect a specific variant and do not present cross-reaction between them, and the final result is given for the internal primer reaction in nested and/or RT-PCR real time, as they were obtained in different samples (Table 5).

In addition, this study showed 100% sensitivity and 100% specificity assessed by nRT-PCR and real-time RT-PCR with SYBR Green. These findings are an early estimate by what is required of a greater number of related studies, increasing the number of samples to obtain better sensitivity and specificity evaluation. However, this assay could be useful, for institutions without access to MAbs and those that have PCR and/or real-time technology as an alternative.

The relevance of the present study falls in the rabies virus typing from original host-brains samples and the association with the variant-specific host performed by nested endpoint PCR or real-time RT-PCR assays. Previous studies report the detection in decomposed brains from dogs and humans samples [32]; in humans exhumed between 8 and 30 days after burial [27]; in wolves by nested RT-PCR [33]; in mice previously infected by heminested RT-PCR [29]; and in bats and herbivores by RT-PCR.

The sequence obtained for this study, a splitting between the urban rabies (dog) and the sylvan rabies (bat, vampire bat, and skunk), was shown in Tables 4 and 5; the results were according to the primers designed associated variant for the dog (urban rabies) and bat, skunk, and vampire bat (sylvan rabies).

## 5. Conclusion

This study describes the development of an alternative tool for RABV typifying in real-time RT-PCR and/or nested RT-PCR, considering dog, skunk, vampire bat, and nonhematophagous bat specific variants.

## Figures and Tables

**Table 1:** RABV N gene sequence for external and internal primer design for different rabies variant.

Nonhematophagous bat			
GI	Host	Country	Collection date

AF351832.1	*Eptesicus fuscus* (big brown bat)		
GU644667.1	*Eptesicus fuscus* (big brown bat)	USA: Michigan	2005
GU644664.1	*Eptesicus fuscus* (big brown bat)	USA: Michigan	2003
GU644662.1	*Eptesicus fuscus* (big brown bat)	USA: Michigan	2003
AY039229.1	*Eptesicus fuscus* (big brown bat)	USA: Adams County, Pennsylvania	1984
AY039228.1	*Eptesicus fuscus* (big brown bat)	USA: El Paso County, Colorado	1985
GU644676.1	*Eptesicus fuscus* (big brown bat)	USA: Virginia	2004
GU644668.1	*Eptesicus fuscus* (big brown bat)	USA: Michigan	2005
AF351862.1	*Eptesicus fuscus* (big brown bat)		
GU644655.1	*Eptesicus fuscus* (big brown bat)	USA: Iowa	2005
GU644695.1	*Eptesicus fuscus* (big brown bat)	USA: Washington	2005
GU644661.1	*Eptesicus fuscus* (big brown bat)	USA: Michigan	2003
AY039227.1	*Eptesicus fuscus* (big brown bat)	USA: Washington	1987
GU644677.1	*Eptesicus fuscus* (big brown bat)	USA: Virginia	2004
GU644670.1	*Eptesicus fuscus* (big brown bat)	USA: Michigan	2005
GU644666.1	*Eptesicus fuscus* (big brown bat)	USA: Michigan	2005
GU644660.1	*Eptesicus fuscus* (big brown bat)	USA: Michigan	2003
GU644656.1	*Eptesicus fuscus* (big brown bat)	USA: Iowa	2005
GU644654.1	*Eptesicus fuscus* (big brown bat)	USA: Iowa	2005
GU644690.1	*Eptesicus fuscus* (big brown bat)	USA: Washington	2004
GU644669.1	*Eptesicus fuscus* (big brown bat)	USA: Michigan	2005
AF351861.1	*Eptesicus fuscus* (big brown bat)		
GU644689.1	*Eptesicus fuscus* (big brown bat)	USA: Washington	2004
GU644684.1	*Eptesicus fuscus* (big brown bat)	USA: Washington	2003
GU644663.1	*Eptesicus fuscus* (big brown bat)	USA: Michigan	2003
GU644659.1	*Eptesicus fuscus* (big brown bat)	USA: Michigan	2003
GU644657.1	*Eptesicus fuscus* (big brown bat)	USA: Michigan	2003
AF351833.1	*Eptesicus fuscus* (big brown bat)		
AF351828.1	*Eptesicus fuscus* (big brown bat)		
GU644665.1	*Eptesicus fuscus* (big brown bat)	USA: Michigan	2005
GU644671.1	*Eptesicus fuscus* (big brown bat)	USA: New Jersey	2005
GU644658.1	*Eptesicus fuscus* (big brown bat)	USA: Michigan	2003
GU644652.1	*Eptesicus fuscus* (big brown bat)	USA: Georgia	2004
AF351831.1	*Eptesicus fuscus* (big brown bat)		
AF351855.1	*Eptesicus fuscus* (big brown bat)		
GU644754.1	*Nycticeius humeralis* (evening bat )	USA: Florida	2001
AF351854.1	*Eptesicus fuscus* (big brown bat)		
AF394868.1	*Antrozous pallidus* (pallid bat)	USA: Monterey, California	1991
AY039225.1	*Myotis austroriparius* (southeastern myotis bat)	USA: Highlands County, Florida	1988
AF351829.1	*Eptesicus fuscus* (big brown bat)		
AF394871.1	*Myotis californicus* (California bat)	USA: Plumas County, California	1987
AF351859.1	*Eptesicus fuscus* (big brown bat)		
AF351860.1	*Eptesicus fuscus* (big brown bat)		
GU644673.1	*Eptesicus fuscus* (big brown bat)	USA: New Jersey	2005
AY039226.1	*Eptesicus fuscus* (big brown bat)	USA: Perry County, Pennsylvania	1984
AF351839.1	*Myotis* sp. (bat)		
AF351853.1	*Eptesicus fuscus* (big brown bat)		
HQ341796.1	*Myotis chiloensis* (bat)	Chile	2009
AF351827.1	*Eptesicus fuscus* (big brown bat)		
GU644675.1	*Eptesicus fuscus* (big brown bat)	USA: New Jersey	2005

Skunk			
GI	Host	Country	Collection date

JQ513553	Skunk V854	Mexico: San Luis Potosí	2002
JQ513552	Skunk V658	USA: Mariposa County, California	1997
JQ513548	Skunk V652	USA: Mariposa County, California	1997
JQ513547	Skunk V651	USA: Mariposa County, California	1997
JQ513551	Fox V657	USA: Mariposa County, California	1997
JQ513541	Dog V640	USA: Sonoma County, California	1994
JQ513546	Skunk	USA: Trinity County, California	1997
JQ513542	Mountain lion	USA: Yolo County, California	1994
JQ513545	Skunk	USA: Mendocino County, California	1997
JQ513549	Skunk	USA: Amador County, California	1997
JQ513539	Skunk	USA: Glenn County, California	1994
JQ513544	Skunk	USA: Colusa County, California	1994
JQ513540	Skunk	USA: Sutter County, California	1994
JQ513550	Skunk	USA: Glenn County, California	1997
FJ228485	Cow	Mexico: Chihuahua	1999
FJ228484	*Spilogale putorius leucoparia*, skunk	Mexico: San Luis Potosí	2002
FJ228483	*Spilogale putorius leucoparia*, skunk	Mexico: Zacatecas	2001
JX856026.1	*Mephitis mephitis* (striped skunk)	USA	2009
JX856036.1	Bovine (cow)	USA	2009
JX856035.1	*Mephitis mephitis* (striped skunk)	USA	2009
JX855972.1	*Felis silvestris* (bobcat)	USA	2009
JX856024.1	*Mephitis mephitis* (striped skunk)	USA	2009
JX856023.1	Bovine (cow)	USA	2009
JX856017.1	Bovine (cow)	USA	2009
JX856016.1	*Mephitis mephitis* (striped skunk)	USA	2009
JX856015.1	*Mephitis mephitis* (striped skunk)	USA	2009
JX856014.1	*Mephitis mephitis* (striped skunk)	USA	2009
JX856006.1	*Mephitis mephitis* (striped skunk)	USA	2009
JX856004.1	*Mephitis mephitis* (striped skunk)	USA	2009
JX856001.1	*Mephitis mephitis* (striped skunk)	USA	2009
JX855993.1	*Mephitis mephitis* (striped skunk)	USA	2009
JX855990.1	*Mephitis mephitis* (striped skunk)	USA	2009
JX855980.1	*Felis catus* (cat)	USA	2009
JX855988.1	*Mephitis mephitis* (striped skunk)	USA	2009
JX855987.1	*Mephitis mephitis* (striped skunk)	USA	2009
JX855986.1	*Mephitis mephitis* (striped skunk)	USA	2009
JX855985.1	*Mephitis mephitis* (striped skunk)	USA	2009
JX855984.1	*Mephitis mephitis* (striped skunk)	USA	2009
JX855981.1	*Mephitis mephitis* (striped skunk)	USA	2009
JX855979.1	*Felis catus* (cat)	USA	2009
JX855973.1	*Mephitis mephitis* (striped skunk)	USA	2009
JX855976.1	*Canis lupus familiaris* (dog)	USA	2009
JX855975.1	*Mephitis mephitis* (striped skunk)	USA	2009
JX855974.1	*Mephitis mephitis* (striped skunk)	USA	2009
JX855970.1	*Mephitis mephitis* (striped skunk)	USA	2009
JX855968.1	*Mephitis mephitis* (striped skunk)	USA	2009
JX855967.1	*Mephitis mephitis* (striped skunk)	USA	2009
JX855966.1	*Mephitis mephitis* (striped skunk)	USA	2009
JX855965.1	*Equus caballus* (horse)	USA	2009
JX855963.1	*Mephitis mephitis* (striped skunk)	USA	2009
JX855962.1	*Mephitis mephitis* (striped skunk)	USA	2009

*Dog *			
GI	Host	Country	Collection date

FJ228513.1	*Canis lupus familiaris* (dog)	Mexico: Estado de México	2000
FJ228512.1	*Canis lupus familiaris* (dog)	Mexico: Estado de México	2002
FJ228507.1	*Canis lupus familiaris* (dog)	Mexico: Estado de México	1999
FJ228532.1	*Canis lupus familiaris* (dog)	Mexico: Puebla	1995
FJ228506.1	*Canis lupus familiaris* (dog)	Mexico: Guerrero	1999
FJ228505.1	*Canis lupus familiaris* (dog)	Mexico: Tlaxcala	2002
FJ228504.1	*Canis lupus familiaris* (dog)	Mexico: Puebla	2001
FJ228503.1	*Canis lupus familiaris* (dog)	Mexico: Tlaxcala	2000
FJ228502.1	*Canis lupus familiaris* (dog)	Mexico: Distrito Federal	1999
KJ001535.1	*Canis lupus familiaris* (dog)	Mexico	2005
KJ001525.1	*Canis lupus familiaris* (dog)	Mexico	2009
KJ001524.1	*Canis lupus familiaris* (dog)	Mexico	2005
KJ001518.1	*Canis lupus familiaris* (dog)	Mexico	2011
KJ001517.1	*Canis lupus familiaris* (dog)	Mexico	2011
KJ001516.1	*Canis lupus familiaris* (dog)	Mexico	2011
KJ001515.1	*Canis lupus familiaris* (dog)	Mexico	2011
KJ001509.1	*Canis lupus familiaris* (dog)	Mexico	2009
KJ001502.1	*Canis lupus familiaris* (dog)	Mexico	2006
KJ001500.1	*Canis lupus familiaris* (dog)	Mexico	2006
KJ001499.1	*Canis lupus familiaris* (dog)	Mexico	2006
KJ001492.1	*Canis lupus familiaris* (dog)	Mexico	2005
KJ001490.1	*Canis lupus familiaris* (dog)	Mexico	2005
KJ001488.1	*Canis lupus familiaris* (dog)	Mexico	2005
FJ228525.1	*Canis lupus familiaris* (dog)	Mexico: Yucatán	2002
FJ228523.1	*Canis lupus familiaris* (dog)	Mexico: Yucatán	1998
FJ228521.1	*Canis lupus familiaris* (dog)	Mexico: Durango	1991
FJ228518.1	*Canis lupus familiaris* (dog)	Mexico: Chiapas	2002
FJ228511.1	*Bos taurus* (cow)	Mexico: Puebla	1994
FJ228510.1	*Sus scrofa domesticus* (pig)	Mexico: Distrito Federal	1991
FJ228509.1	*Mustela putorius furo* (ferret)	Mexico: Distrito Federal	1990
FJ228508.1	*Homo sapiens* (human)	Mexico: Distrito Federal	1991
FJ228522.1	*Bos taurus *(cow)	Mexico: Chihuahua	1994
FJ228519.1	*Felis catus* (cat)	Mexico: Michoacán	1990
AY854591.1	*Canis lupus familiaris* (dog)	Mexico	
AY854589.1	*Canis lupus familiaris* (dog)	Mexico	
FJ228526.1	*Canis latrans* (coyote)	Mexico: Coahuila	2001

*Vampire bat*			
GU991828.1	Desmodontinae (vampire bat V3)		
GU991827.1	Desmodontinae (vampire bat V3)	Mexico: East Mexico	1999
GU991826.1	Desmodontinae (vampire bat V3)	Mexico: East Mexico	1999
GU991825.1	Desmodontinae (vampire bat V3)	Mexico: East Mexico	2004
GU991824.1	Desmodontinae (vampire bat V11)	Mexico: East Mexico	2002
GU991823.1	Desmodontinae (vampire bat V11)	East Mexico	2003
KP202393.1	Desmodontinae (vampire bat)	Mexico	1988
AY854592.1	Desmodontinae (vampire bat)	Mexico	
AY854587.1	Desmodontinae (vampire bat)	Mexico	
AY854595.1	Desmodontinae (vampire bat)	Mexico	
AY854594.1	Desmodontinae (vampire bat)	Mexico	
FJ228491.1	*Bos taurus* (cow)	Mexico: Tamaulipas	2003
FJ228490.1	*Bos taurus* (cow)	Mexico: Veracruz	2003
FJ228489.1	*Ovis aries* (sheep)	Mexico: Hidalgo	2003
FJ228488.1	*Bos taurus* (cow)	Mexico: San Luis Potosí	2004
AY877435.1	Desmodontinae (vampire bat)		
AY877434.1	Desmodontinae (vampire bat)		
AY877433.1	Desmodontinae (vampire bat)		

**Table 2:** RABV isolated typed by MAbs used in the present study.

Sample	Sample name	Host species	Antigenic variant
1	68EDOMEXDOG05	Dog 068	V1
2	647EDOMEXDOG05	Dog 647	V1
3	659EDOMEXDOG05	Dog 659	V1
4	748EDOMEXDOG05	Dog 748	V1
5	2293EDOMEXDOG05	Dog 2293	V1
6	885EDOMEXDOG05	Dog 885	V1
7	658EDOMEXCOW05	Cow 658	V8
8	460EDOMEXCOW11	Cow 460	V8
9	757EDOMEXMUR06	Bat nonhematophagous 757	A
10	1594EDOMEXVAM07	Bat nonhematophagous 1594	V5
11	1079EDOMEXMUR08	Bat nonhematophagous 1079	None
12	3919EDOMEXVAM05	Vampire bat 3919	None
13	110EDOMEXVAM06	Vampire bat 110	Atypical
14	1369EDOMEXSK06	Skunk 1369	V8
15	65EDOMEXDOG05	Dog	NA
16	543EDOMEXDOG05	Dog	NA
17	642EDOMEXDOG05	Dog	NA
18	223EDOMEXDOG05	Skunk	NA
19	1001EDOMEXDOG05	Skunk	NA
20	455EDOMEXDOG05	Cow	NA
21	755EDOMEXDOG05	Bat nonhematophagous	NA
22	2187EDOMEXDOG05	Vampire bat	NA
23	Negative control	CN	NA

All samples are from Mexican state. Varian antigenic test: V1, dog; V5,* Tadarida brasiliensis*; V8, skunk; V11, vampire bat; A, atypical; NA, not applicable; AC Number: sequences refer to NCBI accession number (http://www.ncbi.nlm.nih.gov/).

**Table 3:** Primer details for host mammals specific rabies virus variants detection in Mexico.

	Primer name	Sequence (3′ to 5′)	Sense	Rabies variant detection	Fragment size (pb)	Tm (°C)	Length^*∗*^ (pb)
E	VAMPIROF	TTCAAGGTCAATAATCAGGTGGTCTCTC	F	Vampire bat	836	59	22–49
	VAMPIRORC	AGACTGCTGTTCCTCATTCCTATTT	R			53.8	833–857
I	FBVFQ	ATTGGGCTCTAACAGGGGGCAT	F		177	59.6	356–377
	FBVRCQ	ATAGAGCAGATTTTCGAGACAGCCCCCT	R			62.5	575–602

E	PERROF	TTCAAAGTCAATAATCAGGTGGTC	F	Dog	1288	51.9	22–45
	PERRORC	AATCATCAAGCCCGTCCAAACT	R			56.4	1288–1309
I	FBPFQ	CAAGAATATGAGGCGGCTGAACT	F		212	55	1099–1120
	PERRORC	AATCATCAAGCCCGTCCAAACT	R			56.4	1288–1309

E	MURCIELAGOF	GACCCTGATGATGTATGCTCTTAT	F	Bat	668	51	196–219
	MURCIELAGORC	GTTCCTCACTCYTATTTCATCCA	R			50.6	742–764
I	FBMFQ	GCTTGACCCTGATGATGTATGCTCTTAT	F		184	59	192–219
	FBMRCQ	TGGGCTCTAACAGGGGGTATGG	R			58.7	358–379

E	ZORRILLOF	ATAGAACAGATTTTTGAGACGGC	F	Skunk	794	51.4	505–527
	ZORRILLORC	TGTCTCAGTTAGTTCCAATCATCAAGC	R			56.9	1272–1298
I	ZORRILLOF	ATAGAACAGATTTTTGAGACGGC	F		359	51.4	506–527
	FBZRCQ	GTTCCTCACTCCTATTTCATCCA	R			51.7	742–764

Nested endpoint PCR and real-time RT-PCR primers designed. E: external; I: internal; F: forward; R: reverse. ^*∗*^According to RABV strain SAD VA1 sequence.

**Table 4:** Comparison between different methods of rabies virus variants detection.

Sample	Host	FAT	Antigenic variant	External RT-PCR				Internal PCR				SYBR Green				GenBank sequence AC number (N gene)
				D	V	B	S	d	v	b	s	*d*	*v*	*b*	*s*	
1	Dog 0068	+	V1	+	−	−	−	+	−	−	−	+	−	−	−	JQ037820
2	Dog 647	+	V1	+	−	−	−	+	−	−	−	+	−	−	−	JQ037819
3	Dog 659	+	V1	+	−	−	−	+	−	−	−	+	−	−	−	JQ037823
4	Dog 748	+	V1	+	−	−	−	+	−	−	−	+	−	−	−	JQ037821
5	Dog 2293	+	V1	+	−	−	−	+	−	−	−	+	−	−	−	JQ037824
6	Dog 885	+	V1	+	−	−	−	+	−	−	−	+	−	−	−	JQ037822
7	Cow 658	+	V8	−	+	−	−	−	+	−	−	−	+	−	−	JQ037825
8	Cow 2688	+	V8	−	+	−	−	−	+	−	−	−	+	−	−	JQ037826
9	Bat nonhematophagous 757	+	A	−	−	+	−	−	−	+	−	−	−	+	−	JQ037830
10	Bat nonhematophagous 1594	+	V5	−	−	+	−	−	−	+	−	−	−	+	−	JQ037829
11	Bat nonhematophagous 1079	+	None	−	−	+	−	−	−	+	−	−	−	+	−	JQ037831
12	Vampire bat 3919	+	None	−	−	+	−	−	+	−	−	−	+	−	−	JQ037827
13	Vampire bat 110	+	Atypical	−	−	+	−	−	+	−	−	−	+	−	−	JQ037828
14	Skunk 1369	+	V8	−	−	−	+	−	−	−	+	−	−	−	+	JQ037818
15	65EDOMEXDOG05	−	NA	−	−	−	−	−	−	−	−	−	−	−	−	—
16	543EDOMEXDOG05	−	NA	−	−	−	−	−	−	−	−	−	−	−	−	—
17	642EDOMEXDOG05	−	NA	−	−	−	−	−	−	−	−	−	−	−	−	—
18	223EDOMEXDOG05	−	NA	−	−	−	−	−	−	−	−	−	−	−	−	—
19	1001EDOMEXDOG05	−	NA	−	−	−	−	−	−	−	−	−	−	−	−	—
20	455EDOMEXDOG05	−	NA	−	−	−	−	−	−	−	−	−	−	−	−	—
21	755EDOMEXDOG05	−	NA	−	−	−	−	−	−	−	−	−	−	−	−	—
22	2187EDOMEXDOG05	−	NA	−	−	−	−	−	−	−	−	−	−	−	−	—
23	Negative control	−	NA	−	−	−	−	−	−	−	−	−	−	−	−	—

FAT: fluorescent antibody test; Varian antigenic test: V1, dog; V5, *Tadarida brasiliensis*; V8, skunk; V11, vampire bat; A, atypical; ^+^positive diagnosis. Capital letter: positive external RT-PCR; lowercased letter: positive internal PCR; italic letter: positive SYBR Green. Variants were represented in the following sense: D, dog; V, vampire; B, bat; and S, skunk. Sequences refer to NCBI accession number (http://www.ncbi.nlm.nih.gov/).

**Table 5:** Test results interpretation of nested RT-PCR.

Rabies variant detection/primer name	VAMPIROF-VAMPIRORC	FBVFQ-FBVRCQ^*∗*,*∗∗*^	PERROF-PERRORC	FBPFQ-PERRORC^*∗*,*∗∗*^	MURCIELAGOF-MURCIELAGORC	FBMFQ-FBMRCQ^*∗*,*∗∗*^	ZORRILLOF-ZORRILLORC	ZORRILLOF-FBZRCQ^*∗*,*∗∗*^
Vampire bat	+	+	−	−	−	−	−	−
Dog	−	−	+	+	−	−	−	−
Bat	−	−	−	−	+	+	−	−
Skunk	−	−	−	−	−	−	+	+

Only a sample can be considered positive if the result with the internal primers is positive regardless of the outcome of the external primers, ^*∗*^for nested and/or ^*∗∗*^real-time RT-PCR.
